# Domestic Violence Against Women in North African and Middle Eastern Countries: A Scoping Review

**DOI:** 10.1177/15248380211036070

**Published:** 2021-08-05

**Authors:** Sezer Kisa, Rusan Gungor, Adnan Kisa

**Affiliations:** 1 Faculty of Health Sciences, Department of Nursing and Health Promotion, Oslo Metropolitan University, Norway; 2 Oslo University, Norway; 3 School of Health Sciences, Kristiania University College, Oslo, Norway

**Keywords:** domestic violence, Middle East, North Africa, women

## Abstract

This scoping review aimed to identify the scope of the current literature on the prevalence, consequences, and risk factors of domestic violence (DV) against women by their husbands or male partners in North African and Middle Eastern countries. The methodology for this scoping review was based on the framework outlined by Arksey and O’Malley. Studies published on DV against women over the age of 15 by partner or husband and published in peer-reviewed scientific journals between January 1970 and April 2018 were included in the review. The databases MEDLINE, PsychINFO, CINAHL, HealthSTAR, EMBASE, Scopus, African Journals Online, Turkish Journal Database, and gray literature sources were searched. On completion of the review process, 151 full-text articles were identified for charting. This review demonstrated that women’s age, women’s education level, duration of marriage, history of childhood abuse/witnessing family violence, living in the rural region, and family income level were negatively associated with DV, indicating that younger women, women with lower education, a longer marriage duration, and a lower income level had a higher risk of exposure to DV in this region. Anxiety, depression/insomnia, and physical injury were the most common health problems reported by victims in the region. The highest proportion of women with no response to violence was reported in Jordan, Saudi Arabia, and Turkey. The findings of this scoping review represent the first attempt to summarize the literature from North African and Middle Eastern countries and demonstrate the similarity in DV-related behaviors among women despite the cultural and regional diversity of the studies.

Violence against women (VAW) is defined as “any act of gender-based violence that results in, or is likely to result in, physical, sexual, or mental harm or suffering to women, including threats of such acts, coercion or arbitrary deprivation of liberty, whether occurring in public or in private life” (United Nations, 1993). It has been established for a long period that VAW is a major obstacle to women’s equality, security, and their right to enjoy basic freedoms. It is a universal problem seen in every country and across all societies regardless of age, class, education, income, religion, ethnicity, and culture. A multicountry study, which included 24,000 women participants, indicated that violence by an intimate male partner was widespread in all the countries, and one in three women experienced physical or sexual violence in their lifetime (World Health Organization, 2005). Similarly, the survey on VAW, in which 42,000 women in 28 European Union countries participated, reported that 34% of women were exposed to physical violence. Furthermore, more than 43% were exposed to some form of psychological violence, 32% to psychologically abusive behavior, and 5% to economic violence in their current relationship (FRA—European Union Agency for Fundamental Rights, 2014).

Recent studies have indicated that men and women have an equal risk of being abusers and victims (Colorado-Yohar et al., 2016; Fawson, 2015; Hamberger & Larsen, 2015). However, factors that increase the likelihood of domestic violence (DV) against women include socio-demographic factors such as age, marital status, age at marriage, number of children, lower socioeconomic status; sociocultural factors such as gender roles, unequal power relations between men and women, the region of residence, women’s religion and occupation; family-related factors such as marriage duration, witnessing violence in the family, history of childhood violence (Saffari et al., 2017; Wachter et al., 2018; Zakaliyat & Susuman, 2018).

VAW continues to be a major public health problem, which may cause women to suffer physical injuries, long-lasting mental health problems such as anxiety, depression, antisocial behavior, suicidal behavior, low self-esteem, social isolation, and an inability to care for themselves and their families, as well as gynecological, gastrointestinal, and cardiovascular problems (Colorado-Yohar et al., 2016; Jack et al., 2018; Kulwicki et al., 2015; Lafta, 2008; Niolon et al., 2017). Furthermore, DV causes loss of healthy life years in women of reproductive age and death (Alhabib et al., 2010; Niolon et al., 2017).

## North Africa (NA) and Middle East (ME)

It is also well established in the literature that VAW in the form of DV, family violence, female genital mutilation, forced/child marriages, and honor killings are common throughout NA and the ME, and despite the high prevalence rates, DV continues to be an underreported problem across this region (Alhalal et al., 2019; Boy & Kulczycki, 2008; Organisation for Economic Co-operation and Development [OECD]/Centre for Arab Women Training and Research [CAWTAR], 2014). More recently, a study from Saudi Arabia highlighted the need to have a comprehensive understanding of VAW to design interventions, allocate resources, and develop reform policies (Alhalal et al., 2019).The majority of the countries in the ME and NA are multiethnic, predominantly masculine and collectivist and have the largest gender gap (Alhalal et al., 2019; Archer, 2006; World Economic Forum, 2021). In masculine societies, women are surrounded by ingrained patriarchal cultural traditions and religious boundaries and often represent cooperation, modesty and weak (Archer, 2006). For example, Saudi Arabia and Iran explicitly include religious rules, which have significant effects on women’s perceptions of DV, in their national laws (Aghtaie, 2016). Turkey has more secular regulations that require woman’s complaints to formal institutions. However, women living in conservative and patriarchal parts of the country in the rural areas face death threats from their husbands when they complain (Akadli Ergocmen et al., 2013). In addition to the cultural and religious boundaries, high female illiteracy, low female labor force participation and political participation, and high poverty rates among women are also widespread in the regions (OECD/CAWTAR, 2014; United Nations Development Program, 2020).

Conservative and pervasive patriarchal gender attitudes significantly affect women’s status and is “consistent with an innate belief in male supremacy, giving men the privilege to discipline women. As stated by Kulczycki and Windle (2011), “nondemocratic regimes view gender equality as a distinctly less important goal than political stability, and economic and other concerns,” indicating that this is a matter of gender equality issue in the ME and NA countries. Consequently, DV against women, early and forced marriages, and honor killings are frequently encountered problems in the ME and NA compared to other parts of the world. However, despite legal protections, DV and family violence are often not penalized and remain taboo due to perceptions about family unity, gender discrimination, and misinterpreted religious beliefs, which support the attitudes that tolerate violent behaviors against women in the family and community. DV is, therefore, hidden and regarded as a private family concern in the region (Alhalal et al., 2019).

In patriarchal societies where gender norms and culture are powerful influences on VAW (Archer, 2006), it is recommended to establish culturally appropriate interventions to prevent violence. However, studies related to DV in NA and the ME have mostly focused on topics such as honor killings, female genital mutilation, and child marriages, and relatively little is known about the frequency of DV against women, its health consequences, and the affecting societal and cultural factors in the region (Alhalal et al., 2019; Kulwicki et al., 2015). Understanding DV and the associated health consequences among these women are vital to increasing their quality of life and overall health, both personally and socially, and helping avoid the economic ruin caused by violence. While numerous studies have investigated DV against women in Western countries, there are little reliable data on DV prevalence against women in NA and the ME. In addition, recent studies have recommended that an effort should be made to address the cultural practices that support violent behaviors and inequality between women and men in the ME. This review will shed light on the magnitude of DV problem, its consequences, the associated risk factors, and women’s responses to violent behaviors in selected NA and the ME countries.

## Method

For the purposes of this study, a scoping review was defined as a type of research synthesis that aims to map the literature on a particular topic or research area and provide an opportunity to identify key concepts; gaps in the research; and types and sources of evidence to inform practice, policy-making, and research. The methodology for this scoping review was based on the framework outlined by Arksey and O’Malley (2005) and the ensuing recommendations made by Levac et al. (2010). The review included the following five key phases: (1) identifying the research question; (2) identifying relevant studies; (3) study selection; (4) charting the data; and (5) collating, summarizing, and reporting the results. The optional “consultation exercise” of the framework was not conducted.

### Research Question

This review was guided by the question, “What are the prevalence, consequences, and risk factors of DV against women by their husbands or male partners in North African and Middle Eastern countries?” In addition, the researchers sought to determine abused women’s responses to DV.

### Data Sources and Search Strategy

A search was conducted on June 29, 2018, for papers published between January 1970 and April 2018 across eight electronic databases: MEDLINE, PsychINFO, CINAHL, HealthSTAR, EMBASE, Scopus, African Journals Online, and Turkish Journal Database as well as gray literature. The databases were selected due to their comprehensiveness and coverage of a broad range of disciplines. Three researchers assisted in conducting the search, and a librarian at the Oslo Metropolitan University provided technical guidance. Algeria, Bahrein, Egypt, Iran, Iraq, Jordan, Kuwait, Lebanon, Libya, Morocco, Sudan, Palestine, Oman, Qatar, Saudi Arabia, Syria, Tunisia, Turkey, the United Arab Emirates (UAE), and Yemen were included in the search. Only studies that had been conducted in the selected countries were included. A few authors were contacted to obtain the full text of their research on DV.

For the purposes of this review, the following search terms were used:

Population: women over 12 years old OR adolescent* OR teen* OR “young adult” OR “women”

Exposure: violence* OR abuse* OR assault* OR “dating violence” OR “domestic violence” OR “family violence” OR “partner violence” OR “intimate partner violence” OR “husband violence” OR “physical abuse” OR “verbal abuse” OR “spousal abuse” OR “gender-based violence” OR “sexual coercion” OR “community violence” OR “sexual violence” OR “sexual coercion” OR batter* OR harassment* OR rape*

Location: “Middle East” OR “North Africa” OR Algeria* OR Bahrein* OR Egypt* OR Iran* OR Iraq* OR Jordan* OR Kuwait* OR Lebanon* OR Libya* OR Morocco* OR Sudan* OR Palestine* OR Oman* OR Qatar* OR Saudi Arabia* OR Syria* Tunisia* OR Turkey* OR United Arab Emirates* OR Yemen*

Outcome: “prevalence” OR “experience” OR “attitude” OR “location of abuse” OR “reaction to abuse” OR “response to abuse”

### Study Selection

**Figure 1.**
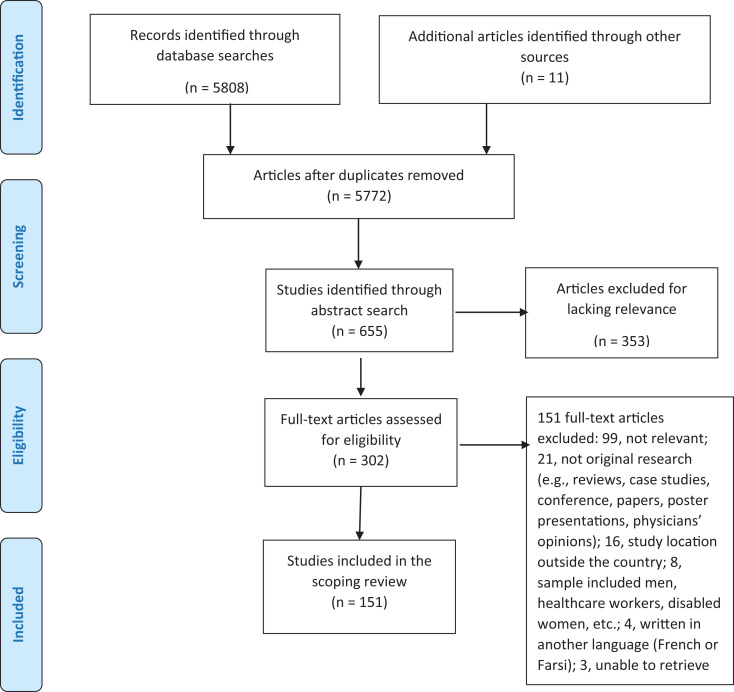
Preferred Reporting Items for Systematic Reviews and Meta-Analyses (PRISMA) flowchart.

As a result of the database search, 5,819 studies were identified, and 5,772 remained after the duplicates had been removed. Using EndNote software (version X9, Clarivate Analytics), as a research tool, all the remaining titles, abstracts, and full-text reviews were assessed by members of the research team using a specific inclusion/exclusion criteria form. Three researchers independently reviewed the full text of the articles for inclusion, and any disagreements were resolved through discussion until consensus was achieved. Figure 1 demonstrates the article review process. On completion of the review process, 151 full-text articles were identified for charting.

### Charting the Data

Using a Microsoft Excel 2010 (Microsoft Corporation, Redmond, WA) spreadsheet, the researchers developed a data charting form to facilitate the data extraction. To help answer the research question, the following data were charted: author/year, title, research location, aim/purpose, method, prevalence and type of violence, risk factors, health consequences caused by violence, and victims’ responses to violence.

### Collating, Summarizing, and Reporting the Results

The final stage of the scoping review provided a descriptive summary and qualitative thematic analysis of the results.

## Results

In this review, 5,808 articles on DV in NA and the ME were identified in the eight databases. Moreover, 11 other studies were identified through a web search and a search of gray literature. Among the 151 studies that fulfilled the inclusion criteria and were included in the study, the majority were cross-sectional descriptive studies, while the remaining studies took the form of mixed methods studies and qualitative studies. We identified four major themes in the reviewed articles. These themes consisted of prevalence and types of DV, risk factors to DV, women’s response to DV, and health consequences.

### Prevalence of Violence

Among the 151 studies, 54 between 2004 and 2017 reported data about the prevalence of DV against women aged between 15 and 88 years in 11 countries (Egypt, Iran, Iraq, Jordan, Lebanon, Palestine, Saudi Arabia, Syria, Tunisia, Turkey, and Yemen), 111 studies reported the prevalence rate of lifetime physical violence, 92 studies reported psychological/verbal violence, 82 studies reported sexual violence, and 28 studies included data on economic violence. The prevalence of DV varied widely across countries. The highest lifetime prevalence for violence was found in Turkey (89.3%), while the highest prevalence rate for violence over the previous 12 months was reported in a survey study in Jordan (98%). The lowest rates of lifetime violence were found in Lebanon (35%), Jordan (50%), and Yemen (54.5%). The highest rates of physical violence were found in Turkey (95.2%) and Lebanon (66%), while the lowest rates were reported in Syria, Palestine, and Tunisia (26.2%, 29.9%, and 32.0%, respectively). The highest rates of sexual violence were reported in Iran (81.5%) and Turkey (74.6%), while the lowest rates were found in Palestine (10.6%), Saudi Arabia (12.7%), Tunisia (10.6%), and Yemen (17.3%). None of the studies in Iran, Iraq, Palestine, or Syria provided data on economic violence. Of the 33 studies reporting economic violence, the highest rates were 78.3% in Turkey, 41.1% in Tunisia, 40.8% in Egypt (one study), 35.1% in Jordan, 34% in Yemen, 33% in Lebanon, and 26% in Saudi Arabia (one study; see Table 1).

**Table 1.** Characteristics of Domestic Violence.

Author (Year)	Country	Study Design	Violence Experience		Type of Domestic Violence				
			Ever/Lifetime Abuse (%)	Abused in Last 12 Months (%)	Physical (%)	Psychological/Verbal (%)	Sexual (%)	Economic (%)	Multiple Types of Violence (%)
USAID (2009)	Egypt	Secondary Data Analysis (EDHS-2005)		19	33		22		
F. Hassan et al. (2004)	Egypt	Cross-sectional descriptive—population-based		10.5	11.1				
Vyas (2017)	Egypt	Cross-sectional descriptive—population-based		14	11.3		0.5		24.8
Ibrahim et al. (2015)	Egypt	Prospective cohort—hospital-based		44.1	15.9	32.6	10		
M. Afifi & von Bothmer (2007)	Egypt	Cross-sectional descriptive—population-based			16.4		3.4		
Habib et al. (2011)	Egypt	Cross-sectional descriptive- Population-based	57.4		29.9	6.6	7.8		13.1
Mamdouh et al. (2012)	Egypt	Cross-sectional descriptive—population-based	77		50.3	71	37.1	40.8	
Diop-Sidibe´ et al. (2006)	Egypt	Secondary data analysis (EDHS-1995)	34	47					
Guimei et al. (2012)	Egypt	Cross-sectional descriptive					36		
Shaikh et al.(2017)	Egypt	Cross-sectional descriptive	66.1						
Yount et al. (2014)	Egypt	Cross-sectional descriptive	67	34		63			54
Yount (2005)	Egypt	Cross-sectional descriptive	27	09					
Yount (2010)	Egypt	Cross-sectional descriptive—population-based	33	18	Yes ^a^				
Jamshidimanesh et al. (2013)	Iran	Cross-sectional descriptive			5	51.3			
Ardabily et al. (2011)	Iran	Cross-sectional descriptive			14	33.8	08		
Khayat et al. (2017)	Iran	Cross-sectional descriptive			18		39		
Moghaddam Hossieni et al. (2017)	Iran	Cross-sectional descriptive—population-based		Yes	23	66.1	30.5		Yes
Salari & Nakhaee (2008)	Iran	Cross-sectional descriptive			25	35			
Garrusi et al. (2008)	Iran	Cross-sectional descriptive			27	36.7	25		
Saffari et al. (2017)	Iran	Prospective cohort—multicenter			28	64	18		
Nouri et al. (2012)	Iran	Cross-sectional descriptive			60	79.7	32.9		
Farrokh-Eslamlou et al. (2014)	Iran	Cross-sectional population-based			10.2	43.5	17.2		
Abdollahi et al. (2015)	Iran	Prospective cohort			14.1				
Jahanfar & Malekzadegan (2007)	Iran	Cross-sectional descriptive			14.6	60.5	23.5		
Faramarzi et al. (2005a)	Iran	Cross-sectional descriptive		Yes	14.8	80.6	43.6		
Faramarzi et al. (2005b)	Iran	Cross-sectional descriptive		Yes	15	81.5	42.4		
Moghaddam Hosseini et al. (2013)	Iran	Cross-sectional descriptive—population-based		78.1	19.1	66.5			47.4
Hajian et al. (2014)	Iran	Cross-sectional descriptive		Yes	19.6	85.5			
Alijani et al. (2018)	Iran	Cross-sectional descriptive			25.9	85.8	28.2		
Mohammadhosseini et al. (2010)	Iran	Cross-sectional descriptive			26.7	53.5	34.7		
Ahmadi et al. (2017)	Iran	Cross-sectional descriptive			27.6	57.1	26.6		3.4
Hajikhani Golchin et al. (2014)	Iran	Cross-sectional analytic			28.2	34.6	3.65		
Sheikhbardsiri et al. (2017)	Iran	Cross-sectional descriptive			29.2	58	10		
Jamali et al. (2017)	Iran	Cross-sectional descriptive			30.9	69.7	28.1		Yes
Mohamadian et al. (2016)	Iran	Cross-sectional descriptive			33.8	54.2	23.7		
Nojomi et al. (2007)	Iran	Cross-sectional descriptive	59		34.3				
Abbaszadeh et al. (2011)	Iran	Cross-sectional descriptive			37	59.7			
Abadi et al. (2012)	Iran	Cross-sectional descriptive			4.8	26	5.5		1.3
Vakili et al. (2010)	Iran	Cross-sectional descriptive			43.7	82.6	30.9		
M. Hassan et al. (2014)	Iran	Cross-sectional descriptive		72.8	44.1	46	30.2		
Z. S. Asadi et al. (2013)	Iran	Cross-sectional descriptive			45.2	76.7	63.8		Yes
Ramezani et al. (2015)	Iran	Cross-sectional descriptive			55.8	81.2	25.3		
Ghahhari et al. (2008)	Iran	Cross-sectional descriptive			73.5	92.2	49.6		
Mohammadkhani et al. (2009)	Iran	Cross-sectional descriptive			80	78.3	41.8		
Masoudzadeh et al. (2015)	Iran	Cross-sectional descriptive			99.2	10	81.5		
Tavoli et al. (2016)	Iran	Qualitative							64.8
Khodakarami et al. (2009)	Iran	Cross-sectional descriptive	21.3						
Motevaliyan et al. (2017)	Iran	Cross-sectional descriptive		86		82.0			
Rabiei & Nikooseresht (2009)	Iran	Cross-sectional analytical	34.2						
Vameghi et al. (2016)	Iran	Cross-sectional analytical	35.3						
Yari et al. (2013)	Iran	Cross-sectional descriptive					32.9		
Adib-Hajbaghery et al. (2015)	Iran	Cross-sectional analytical			Yes	Yes	Yes	Yes	
AhmadzadAsl et al. (2013)	Iran	Cross-sectional descriptive—population-based		18.9	Yes				
Banaei et al. (2016)	Iran	Cross-sectional analytical		54	33	45	28		
Ghodrati et al. (2017)	Iran	Cross-sectional analytic			Yes	Yes	Yes	Yes	
Malik et al. (2017)	Iraq	Cross-sectional descriptive		72	39	61	18.3		
Al-Tawil (2012)	Iraq	Cross-sectional descriptive			17.6	32.4	9.4		
Al-Atrushi et al. (2013)	Iraq	Cross-sectional descriptive	58.6	45.3	38.9	52.6	21.1		
Clark et al. (2008)	Jordan	Mixed method			31		20		
Spencer et al. (2014)	Jordan	Mixed method			43	61	46		
Oweis et al. (2010)	Jordan	Cross-sectional descriptive	3.4		10.4	47.1	5.7		
Al-Natour et al. (2014)	Jordan	Cross-sectional descriptive			12.5	59	5.1		
Al-Nsour et al. (2009)	Jordan	Cross-sectional descriptive		87	19.6	47.5			
Safadi et al. (2018)	Jordan	Cross-sectional descriptive			22.8		9.6	35.1	22.7
Al-Modallal (2016a)	Jordan	Cross-sectional descriptive			29.9				
Clark et al. (2009)	Jordan	Qualitative			31.2	50.2	18.8		
Al-Modallal (2016b)	Jordan	Cross-sectional descriptive	31.2		36.1		19.6		11.8
Al-Badayneh (2012)	Jordan	Cross-sectional descriptive		98					
Al-Modallal et al. (2012)	Jordan	Cross-sectional descriptive	50			38.4			
Clark et al. (2012)	Jordan	Mixed method	40						23
Haddad et al. (2011)	Jordan	Prospective cohort	30				06		
Okour & Badernah (2011)	Jordan	Cross-sectional descriptive	3.4	40.9	34.7	28.1	15.5		
Usta et al. (2007)	Lebanon	Cross-sectional descriptive	35		66	88		12	
Awwad et al. (2014)	Lebanon	Cross-sectional descriptive			40.7	83.5	33	33.0	
HajYahia & Clark. (2013)	Palestine	Cross-sectional descriptive			22.2	61.6	10.6		
Sousa et al. (2015)	Palestine	Cross-sectional descriptive		50	29.9	38			
Barnawi (2017)	Saudi Arabia	Cross-sectional descriptive		20	20	69	10	26	
Halawi Azhar et al. (2017)	Saudi Arabia	Cross-sectional descriptive	36		32				
Al Dosary (2016)	Saudi Arabia	Cross-sectional descriptive			57	75.1			
Fageeh (2014)	Saudi Arabia	Cross-sectional descriptive	34		11.6	29	4.8		
Al-Faris et al. (2013)	Saudi Arabia	Cross-sectional descriptive	Yes		12.2				
Z. E. M Afifi et al. (2011)	Saudi Arabia	Cross-sectional descriptive—population-based	39.3		17.9	35.9	6.9		
Tashkandi & Rasheed (2009)	Saudi Arabia	Cross-sectional descriptive	57.7	58.5	25.7	32.8			Yes
Eldoseri & Sharps (2017)	Saudi Arabia	Cross-sectional descriptive	44.5	16	45.5				
Bohlaiga et al. (2014)	Saudi Arabia	Cross-sectional descriptive	18.7		7.5	43.3			49.2
Alquaiz et al. (2017)	Saudi Arabia	Cross-sectional descriptive	43		9	22	12.7		Yes
Alzahrani et al. (2016)	Saudi Arabia	Cross-sectional descriptive		Yes					
Jradi & Abouabbas (2017)	Saudi Arabia	Cross-sectional descriptive		16.5					
Maziak & Asfar (2003)	Syria	Cross-sectional descriptive			26.2				
Anes Jellali et al. (2014)	Tunisia	Cross-sectional descriptive	56.9		32	56.9	10.6	41.1	36
Selek et al. (2012)	Turkey	Cross-sectional descriptive			18	18	18	46	Yes
Korkmaz et al. (2016)	Turkey	Cross-sectional descriptive	Yes		45	68	11	39	
Vahip & Doganavsargil (2006)	Turkey	Cross-sectional descriptive	Yes	Yes	62				
Agcay et al. (2015)	Turkey	Cross-sectional descriptive			72	97.2	62.9		30
Nur (2014)	Turkey	Cross-sectional descriptive—population-based			10		6.2		
Nur (2012)	Turkey	Cross-sectional descriptive—population-based	34.2	5.7	10.3	Yes	6.8		
Alan et al. (2016)	Turkey	Cross-sectional descriptive	39.8		11.5	54.3	12.7	9.3	
Bulut et al. (2017)	Turkey	Cross-sectional descriptive			14.1	48.4	3.1	3.1	
Alper et al. (2005)	Turkey	Cross-sectional descriptive	58.7		14.5				45.5
Ozpinar et al. (2016)	Turkey	Cross-sectional descriptive	Yes	Yes	14.8	20.2	7.9	11.2	
Yüksel Kaptanoğlu et al. (2012)	Turkey	Cross-sectional descriptive	15	Yes	15.1		15.1		
Bilgin Sahin & Erbay Dundar (2017)	Turkey	Cross-sectional descriptive	27.2	13.6	23.3	39.4	9.8	24.4	
Alan et al. (2016)	Turkey	Cross-sectional descriptive			23.9	71.6	13.5	13.5	
Bolu et al. (2015)	Turkey	Cross-sectional descriptive			26.5	57.9	11.2	30.3	Yes
Baran & Gumus (2017)	Turkey	Cross-sectional descriptive			26.6	79.7	11.4	11.4	
Karakoc et al. (2015)	Turkey	Structured clinical interview	Yes		26.7	33.3	20	10	
Deveci et al. (2007)	Turkey	Cross-sectional descriptive		Yes	28.9	49.8	10.8		Yes
Akar et al. (2010)	Turkey	Cross-sectional descriptive	77.9	Yes	29.9	59.6	31.0	60.4	
Öyekçin et al. (2012)	Turkey	Cross-sectional descriptive	Yes		30.4	54.5	6.3	19.3	
Ozturk et al. (2017)	Turkey	Cross-sectional descriptive	Yes		31.9	60.5			
Ergin et al. (2005)	Turkey	Cross-sectional descriptive			34.1	15.8		8.2	29.5
Kocacik & Dogan (2006)	Turkey	Cross-sectional descriptive	Yes		38.3	53.8			
Alaman & Yildiz (2014)	Turkey	Cross-sectional descriptive	Yes		41	50	53		
Mayda & Akkus (2004)	Turkey	Cross-sectional descriptive	Yes		41.4	25.9	8.6		
Kivrak et al. (2015)	Turkey	Cross-sectional descriptive	89.3		54.1	75.4	74.6	78.3	
Sen & Bolsoy (2017)	Turkey	Cross-sectional descriptive	Yes		54.8	61.8	15.9	66.2	
Sahin & Sahin (2003)	Turkey	Cross-sectional descriptive			55.1	44.9			
Akyazi et al. (2018)	Turkey	Cross-sectional descriptive			67.8	0,48	44.1	52.5	
Guciz Dogan et al. (2010)	Turkey	Cross-sectional descriptive			7.8				6.8
Arslantas et al. (2012)	Turkey	Cross-sectional descriptive	24.1		75.8	21			3.2
Karaoglu et al. (2006)	Turkey	Cross-sectional descriptive			8.1	26.7	9.7		
Ersoy & Yildiz (2011)	Turkey	Cross-sectional descriptive			83.1	87.7	60.0	50.8	Yes
Kotan et al. (2017)	Turkey	Cross-sectional descriptive			85.3	10	41.7	52.1	
Izmirli et al. (2014)	Turkey	Cross-sectional descriptive	67.7		88.6	46.5	73.7	31.5	
Yanikkerem et al. (2006)	Turkey	Cross-sectional descriptive—population-based			9.7		36.4		
Uskun et al. (2012)	Turkey	Cross sectional descriptive		Yes	95.2				
Akadli Ergocmen et al. (2013)	Turkey	Cross-sectional descriptive	36.0	Yes	Yes	Yes			
Ergönen et al. (2009)	Turkey	Cross-sectional descriptive	Yes		Yes	Yes	3.4	4.3	
Kavak et al. (2018)	Turkey	Cross-sectional descriptive			Yes	Yes	Yes	Yes	
Kocacik et al. (2007)	Turkey	Cross-sectional descriptive	Yes		Yes	Yes	Yes	Yes	
Marshall & Furr (2010)	Turkey	Secondary data analysis			Yes	Yes	Yes	Yes	
Tetikcok et al. (2016)	Turkey	Secondary data analysis	Yes	Yes	Yes	Yes	Yes	Yes	
Tokuc et al. (2010)	Turkey	Cross-sectional descriptive		34	Yes	93	Yes		
Topbas et al. (2008)	Turkey	Cross-sectional descriptive		Yes	Yes	Yes	Yes	Yes	1.6
Turk et al. (2017)	Turkey	Mixed method			Yes	45.8	Yes		Yes

*Note*. EDHS = Egyptian Demographic Health Survey.

^a^ No specific information on prevalence of domestic violence.

### Risk Factors for DV

The following were reported as risk factors for DV against women in NA and the ME: age, education, occupation, employment status, larger family size, number of children, history of childhood abuse, drug or substance abuse/alcohol use among husband, witnessing family violence, lower socioeconomic status, divorce or separation, smoking status, duration of marriage, age at marriage, marriage without woman’s permission, poor mental health status, wide age difference between couples, female circumcision, and place of residency (urban or rural). Most of the studies reported that women’s age, women’s education level, duration of marriage, and family income level were negatively associated with DV, indicating that younger women, women with lower education, a longer duration of marriage, and a lower income level had a higher risk of exposure to DV in this region. In contrast, some studies found that the risk of DV was higher among older women, women with higher education, and a shorter marriage duration. Few studies reported no difference in violence rates by age, the education levels of women and men, women’s occupation, employment status, family size, socioeconomic status, and marriage duration. High rates of childhood abuse as a risk factor for DV were reported in Turkey, Iran, Saudi Arabia, Egypt, and Jordan and witnessed family violence reported in Iran, Lebanon, Turkey, Egypt, Jordan, Saudi Arabia. Religion was found to be a positively associated determinant for DV, while endogamous marriage, having a co-wife, and a male child preference were reported as negatively associated determinants for DV in seven countries (Egypt, Iran, Iraq, Jordan, Saudi Arabia, Syria, and Turkey). Studies in three countries (Turkey, Lebanon, and Egypt) found that acceptance of violence by women was a risk factor for DV. Female circumcision was reported to be a risk factor for DV in only two countries (Egypt and Iran; see Table 2).

**Table 2.** Risk Factors for Domestic Violence.

Author (Year), Country	Age	Education	Husband With Low Education	Occupation Housewife	Woman Employed	Unemployed Husband	Large Family	No. of Children, Preference for Male Children	History of Childhood Abuse/Witnessing Family Violence	Husband Drug or Substance Abuse/Alcohol Use	Socioeconomic Status	Divorce or Separation	Smoking Husband/Woman	Duration of Marriage, Endogamous Marriage	Marriage Age, Female Circumcision	Marriage Without Permission of Woman	Poor Mental Health	Wide Age Difference Between Couple	Place of Residence	Religion	Having a Co-Wife	Acceptance of Wife of DV
Bohlaiga et al. (2014), Saudi Arabia	Old	Low	+			+	NE	High		+	Low	+	+									
Halawi Azhar etal., 2017, Saudi Arabia		Low	+	+		+				+	Low	+									+	
Banaei et al. (2016), Iran	Young	Low		+										Short								
Akyazi et al. (2018), Turkey	NE	Low			+		+	+	WFV	+		+		EM		+	+					
Mamdouh et al. (2012), Egypt		High	+				+			+		+	+	+	+							
Eldoseri & Sharps (2017), Saudi Arabia	Young						+		HCA	+		+										
Alquaiz et al. (2017), Saudi Arabia	Young		+									+		Long							+	
Ba Obaid & Bijleveld (2002), Yemen	Old	Low										+										
Alper et al (2005), Turkey			+		NE					+	Low	+										
Guimei et al. (2012), Egypt				+			+	+	HCA/WFV	+	Low											+
Maziak & Asfar (2003), Syria		Low	+								Low		+	+	+		+	+	R	+	+	
Jeyaseelan et al. (2004), Egypt	Old	NE	NE	NE					WFV	+	Low											
Arslantas et al. (2012), Turkey		Low/high							HCA	+	Low					+						
Habib et al. (2011), Egypt		Low	+	+				+		+	Low				+							
Yount (2010), Egypt	Young	High							HCA		Low				+/FC							
Yount (2005), Egypt	Young										Low				+							
Barnawi (2017), Saudi Arabia	Young		+					+			Low		+	+								
Hajian et al. (2014), Iran	Young		+							+	Low		+									
Ergin et al. (2005), Turkey		Low	+	+				+	HCA	+	Low											
HajYahia & Clark (2013), Palestine	NE	NE	+			+	+				Low											
Saffari et al. (2017), Iran		High					+		WFV	+	Low											
Kavak et al. (2018), Turkey	Young							High			Low			Long								
Abadi et al. (2012), Iran	Young										Low			Short								
Khodakarami et al. (2009), Iran		Low									Low											
Alzahrani et al. (2016), Saudi Arabia			+			+			HCA	+	Low											
Akadli Ergocmen et al. (2013), Turkey	Old	Low							HCA		Low											
Ibrahim et al. (2015), Egypt	Young	Low						+		+	Low							+				
Al-Faris et al. (2013), Saudi Arabia						+			HCA/WFV	NE	NE		NE									
Usta et al. (2007), Lebanon	NE	Low		+					HCA		NE											
Abujilban et al. (2017), Jordan	Old	NE	NE		NE	NE		NE			NE											
Awwad et al. (2014), Lebanon								+		+			+									+
Tokuc et al. (2010), Turkey	Young/old	Low		+			+															+
Kivrak et al. (2015), Turkey			NE	+	+			+	WFV					NE	+	+	+	+				
Ozer et al. (2015), Turkey																	+					
Kotan et al. (2017), Turkey									HCA								+					
Ghahhari et al. (2008), Iran	Young		+			+		+		+							+					
Garrusi et al. (2008), Iran					+												+					
Dogan et al. (2010), Turkey	Young	Low								+					Young	+						
Baran & Gumus (2017), Turkey	Young													EM	+	+						
Karaoglu et al. (2006), Turkey			+			+		+					+	+		+						
Bolu et al. (2015), Turkey				+	+					+						+						
Yari et al. (2013), Iran															FC	+						
Ardabily et al. (2011), Iran			+			+										+						
Sheikhbardsiri et al. (2017), Iran									HCA	+						+		+				
Bilgin Sahin & Erbay Dundar (2017), Turkey								+	HCA/WFV	+						+						
Al-Tawil (2012), Iraq					NE					+						+					+	
Safadi et al. (2018), Jordan	Old	Low				+									Young				U			
Nouri et al. (2012), Iran					+					+			+		+							
Al-Nsour et al. (2009), Jordan	Old			+											+				R			
Ogulmus & Keskin (2017), Turkey									HCA/WFV	+					+							
Agcay et al. (2015), Turkey	Young	Low						+							+			+	R			
Sen & Bolsoy (2017), Turkey	Old	Low			+				HCA						+						+	
Demir (2017), Turkey	Young														+			+		+		
Hajikhani Golchin et al. (2014), Iran						+			HCA	+			+	+/EM								
Fageeh (2014), Saudi Arabia	Old		+					+					+									
Alan et al. (2016), Turkey			+							+			+									
Al Dosary (2016), Saudi Arabia				+																		
Refaat et al. (2001), Egypt		Low	+	+											FC							
Kocacik & Dogan (2006), Turkey				+					HCA													
Nur (2012), Turkey		Low					+															
Akar et al. (2010), Turkey	Middle age	Low	+	+	+	+	+		HCA/WFV	+												
Clark et al. (2008), Jordan	Old	NE	NE		NE	NE	+	NE										+			+	
Farrokh-Eslamlou et al. (2014), Iran			+			+								+								
Akyuz et al. (2008), Turkey	Young	Low												+								
Pournaghash-Tehrani et al. (2009), Iran																				+		
Oyekcin et al. (2012), Turkey									HCA													
Uskun et al. (2012), Turkey									HCA													
Ahmadi et al. (2017), Iran		Low				+				+												
Alijani et al. (2018), Iran	Young																					
Faramarzi et al. (2005), Iran		Low																	R			
M. Hassan et al. (2014), Iran	Young		+			+																
Jamali et al. (2017), Iran		Low			+																	
Ozpinar et al. (2016), Turkey		Low							HCA													
Izmirli et al. (2014), Turkey																			R			
Abbaszadeh et al. (2011), Iran										+												
Yüksel Kaptanoğlu et al. (2012), Turkey									HCA													
Motevaliyan et al. (2017), Iran									HCA													
Mayda & Akkus (2004), Turkey		Low	+																			
Rabiei & Nikooseresht (2009), Iran									WFV	+												
Korkmaz et al. (2016), Turkey		High							HCA													
Shiraz (2016), Saudi Arabia		Low																				
Mohamadian et al. (2016), Iran	Young	Low			+	+																
Moghaddam Hosseini et al. (2013), Iran		High								+												
Nur (2014), Turkey		Low						+														
Al-Badayneh (2012), Jordan		Low	NE		+	NE			HCA/WFV													
Alan et al. (2016), Turkey									HCA/WFV													
Ergönen et al (2009), Turkey			+						HCA	+												
Deveci et al. (2007), Turkey								+	HCA	+												
Vahip & Doganavsargil (2006), Turkey									HCA													
USAID (2009), Egypt		Low	+		+														U			
Tetikcok et al. (2016), Turkey																			R			
Karakoc et al. (2015), Turkey									HCA/WFV													
Shaikh et al. (2017), Egypt									HCA/WFV													
Al-Modallal (2016), Jordan									HCA/WFV													
Selek et al. (2012), Turkey									HCA													
Okour & Badernah (2011), Jordan								+/PMC											U			
Güleç Öyekçin et al. (2012), Turkey									HCA/WFV					Long								
Kocacik et al. (2007), Turkey				+				+														

*Note*. EM = endogamous marriage; FC = female circumcision; HCA = history of childhood abuse; NE = no effect; PMC = preference for male children; R = rural; U = urban; WFV = witnessing family violence.

### Health Problems Due to Violence

A number of health problems due to DV were identified in the reviewed studies. The majority of the studies reported mental health problems including anxiety and stress (Ahmadzad-Asl et al., 2016; Al-Modallal et al., 2012; Al-Faris et al., 2013; Alizadeh et al., 2015; Akyazi et al., 2018; Anes Jellali et al., 2014; Ba Obaid & Bijleveld, 2002; Barnawi, 2017; Ghodrati et al., 2017; Hajian et al., 2014; Karakoc et al., 2015; Khayat et al., 2017; Kotan et al., 2017; Ozer et al., 2015; Shaikh et al., 2017; Sousa et al., 2015; Vameghi et al., 2016; Yount et al., 2014), depression/insomnia (Abbaszadeh et al., 2011; Ahmadzad-Asl et al., 2016; Akyazi et al., 2018; Alizadeh et al., 2015; Al-Modallal et al., 2012; Anes Jellali et al., 2014; Barnawi, 2017; Bulut et al., 2017; Dolatian et al., 2010; Ersoy & Yildiz, 2011; Jamali et al., 2017; Karakoc et al., 2015; Khayat et al., 2017; Kivrak et al., 2015; Korkmaz et al., 2016; Kotan et al., 2017; Ozer et al., 2015; Vameghi et al., 2016; Yanikkerem et al., 2006), and physical injury (Akadli Ergocmen et al., 2013; Al-Atrushi et al., 2013; Al-Faris et al., 2013; Ardabily et al., 2011; S. Asadi et al., 2017; Awwad et al., 2014; Barnawi, 2017; Ba Obaid & Bijleveld, 2002; Bilgin Sahin & Erbay Dundar, 2017; Damra et al., 2015; Eldoseri & Sharps, 2017; Guimei et al., 2012; Nojomi et al., 2007; Tashkandi & Rasheed, 2009; Yount et al., 2014). Notwithstanding, few studies found increased contraceptive use (Diop-Sidibe´ et al., 2006), fewer visits to antenatal care (Alan et al., 2016; Barnawi, 2017; Diop-Sidibe´ et al., 2006; Ghodrati et al., 2017; Yanikkerem et al., 2006), suicide (Akyazi et al., 2018; Karakoc et al., 2015; Korkmaz et al., 2016; Vizcarra et al., 2004), threatened abortion (Ibrahim et al., 2015), preterm labor (Ersoy & Yildiz, 2011; M. Hassan et al., 2014; Ibrahim et al., 2015; Jamshidimanesh et al., 2013), premature rupture of membranes (Abdollahi et al., 2015; Ibrahim et al., 2015), dystocia (Ibrahim et al., 2015; Khodakarami et al., 2009), fetal distress and fetal death (Ibrahim et al., 2015), low birth weight/low maternal weight (Abdollahi et al., 2015; Abujilban et al., 2017; Ibrahim et al., 2015; Khodakarami et al., 2009), sexually transmitted infections (Ersoy & Yildiz, 2011; Vyas, 2017), and decreased sexual desire/sexual satisfaction (Alaman & Yildiz, 2014; Anes Jellali et al., 2014; Ersoy & Yildiz, 2011; Selek et al., 2012) as health problems due to DV (see Table 3).

**Table 3.** Women’s Responses and Health Consequences of Violence.

Author (Year)	Country	Study Design	Response to Violence							Health consequences			
			Did Not Talk to Anyone/Did Not Seek Help	Reported to Police	Reported to Local Leaders/Family Members/Friends	Reported to Religious Leader	Reported to Health Care Provider	Sought Legal Advice	Located Shelters	Physical Injury	Pregnancy Related ^a^	Mental Health ^b^	Sexuality Related ^c^
Diop-Sidibe´ et al. (2006)	Egypt	Secondary Data Analysis (EDHS-1995)	+								+		
Vyas (2017)	Egypt	Cross-sectional descriptive—population-based											+
Ibrahim et al. (2015)	Egypt	Cross-sectional descriptive—hospital-based									+		
Shaikh et al. (2017)	Egypt	Cross-sectional descriptive										+	
Guimei et al. (2012)	Egypt	Cross-sectional descriptive	+							+			
Yount (2005)	Egypt	Cross-sectional descriptive	+										
Yount et al. (2014)	Egypt	Cross-sectional descriptive								+		+	
Vizcarra et al. (2004)	Egypt	Cross-sectional descriptive										+	
Ardabily et al. (2011)	Iran	Cross-sectional descriptive								+			
Khayat et al. (2017)	Iran	Cross-sectional descriptive	+							+	+	+	
Jamshidimanesh et al. (2013)	Iran	Cross-sectional descriptive									+		
Banaei et al. (2016)	Iran	Cross-sectional analytical					+						
Abdollahi et al. (2015)	Iran	Prospective cohort									+		
Jamali et al. (2017)	Iran	Cross-sectional descriptive										+	
Faramarzi et al. (2005)	Iran	Cross-sectional descriptive					+						
Hajian et al. (2014)	Iran	Cross-sectional descriptive			+							+	
Z. S. Asadi et al. (2013)	Iran	Cross-sectional descriptive					+			+			
Aghakhani et al. (2015)	Iran	Cross-sectional descriptive					+						
Alizadeh et al. (2015)	Iran	Cross-sectional descriptive					+					+	
Nojomi et al. (2007)	Iran	Cross-sectional descriptive								+			
Abbaszadeh et al. (2011)	Iran	Cross-sectional descriptive										+	
M. Hassan et al. (2014)	Iran	Cross-sectional descriptive									+		
Dolatian et al. (2010)	Iran	Cross-sectional descriptive										+	
Khodakarami et al. (2009)	Iran	Cross-sectional descriptive									+		
Vameghi et al. (2016)	Iran	Cross-sectional analytical											
Ahmadzad-Asl et al. (2016)	Iran	Cross-sectional descriptive—population-based										+	
Ghodrati et al. (2017)	Iran	Cross-sectional analytical									+	+	
Malik et al., 2017	Iraq	Cross-sectional descriptive	+	+								+	
Al-Atrushi et al., 2013	Iraq	Cross-sectional descriptive								+			
Spencer et al. (2014)	Jordan	Mixed method	+	+	+	+		+	+				
Haddad et al. (2011)	Jordan	Prospective cohort	+										
Damra et al. (2015)	Jordan	Qualitative					+						
Abujilban et al. (2017)	Jordan	Cross-sectional descriptive									+		
Al-Modallal et al. (2012)	Jordan	Cross-sectional descriptive										+	
Usta et al. (2007)	Lebanon	Cross-sectional descriptive	+		+								
Awwad et al. (2014)	Lebanon	Cross-sectional descriptive					+			+			
Sousa et al. (2015)	Palestine	Cross-sectional descriptive										+	
Barnawi (2017)	Saudi Arabia	Cross-sectional descriptive	+	+			+	+	+	+		+	
Al Dosary (2016)	Saudi Arabia	Cross-sectional descriptive					+					+	
Al-Faris et al. (2013)	Saudi Arabia	Cross-sectional descriptive								+		+	
Fageeh (2014)	Saudi Arabia	Cross-sectional descriptive			+								
Z. E. M. Afifi et al. (2011)	Saudi Arabia	Cross-sectional descriptive—community-based	+		+								
Tashkandi & Rasheed (2009)	Saudi Arabia	Cross-sectional descriptive					+			+			
Eldoseri & Sharps (2017)	Saudi Arabia	Cross-sectional descriptive					+			+			
Jradi & Abouabbas (2017)	Saudi Arabia	Cross-sectional descriptive										+	
Bohlaiga et al. (2014)	Saudi Arabia	Cross-sectional descriptive	+	+	+								
Alzahrani et al. (2016)	Saudi Arabia	Cross-sectional descriptive		+	+								
Anes Jellali et al. (2014)	Tunisia	Cross-sectional descriptive										+	+
Selek et al. (2012)	Turkey	Cross-sectional descriptive											+
Korkmaz et al. (2016)	Turkey	Cross-sectional descriptive										+	
Bulut et al. (2017)	Turkey	Cross-sectional descriptive										+	
Agcay et al. (2015)	Turkey	Cross-sectional descriptive		+									
Alan et al. (2016)	Turkey	Cross-sectional descriptive		+			+	+			+		
Karakoc et al. (2015)	Turkey	Structured clinical interview										+	
Kivrak et al. (2015)	Turkey	Cross-sectional descriptive										+	
Alper et al. (2005)	Turkey	Cross-sectional descriptive					+						
Bilgin Sahin & Erbay Dundar (2017)	Turkey	Cross-sectional descriptive	+	+						+			
Alan et al. (2016)	Turkey	Cross-sectional descriptive		+			+	+	+				
Bolu et al. (2015)	Turkey	Cross-sectional descriptive					+						
Baran & Gumus (2017)	Turkey	Cross-sectional descriptive					+						
Akar et al. (2010)	Turkey	Cross-sectional descriptive					+						
Ergin et al. (2005)	Turkey	Cross-sectional descriptive			+								
Alaman & Yildiz (2014)	Turkey	Cross-sectional descriptive	+	+			+						
Sahin & Sahin (2003)	Turkey	Cross-sectional descriptive		+									
Akyazi et al. (2018)	Turkey	Cross-sectional descriptive							+			+	
Dogan et al. (2010)	Turkey	Cross-sectional descriptive					+						
Karaoglu et al. (2006)	Turkey	Cross-sectional descriptive			+		+						
Ersoy & Yildiz (2011)	Turkey	Cross-sectional descriptive		+	+				+			+	+
Kotan et al. (2017)	Turkey	Cross-sectional descriptive	+		+							+	
Yanikkerem et al. (2006)	Turkey	Cross-sectional descriptive—population-based					+				+	+	
Ogulmus & Keskin (2017)	Turkey	Cross-sectional descriptive		+	+		+		+				
Salcioglu et al. (2017)	Turkey	Qualitative							+				
Akadli Ergocmen et al. (2013)	Turkey	Cross-sectional descriptive		+			+	+		+			
Tokuc et al. (2010)	Turkey	Cross-sectional descriptive					+						
Ozer et al. (2015)	Turkey	Cross-sectional descriptive										+	
Ba Obaid & Bijleveld (2002)	Yemen	Cross-sectional descriptive	+	+						+		+	

*Note.* EDHS **=** Egyptian Demographic Health Survey.

^a^ Pregnancy-related consequences: increased contraceptive use, fewer visits to antenatal care, threatened abortion, preterm labor, premature rupture of membranes, dystocia, fetal distress, fetal death, low birth weight/low maternal weight, caring behavior.

**^b^** Mental health consequences: anxiety, stress, depression, insomnia, suicide.

**^c^** Sexuality-related consequences: sexually transmitted infection, decreased sexual desire/sexual satisfaction.

### Responses to Violence

The women’s responses to DV were identified in 79 studies. These included no response/did not seek help (15 studies), reported to police (15 studies), reported to local leaders/family members/friends (12 studies), reported to a religious leader (one study), reported to healthcare professionals (24 studies), received legal advice (five studies), and went to shelters (seven studies). The highest proportion of women with no response was reported in Jordan (60%–90%), Saudi Arabia (40%–50%), and Turkey (50%). Among all countries, few studies (one study in Saudi Arabia and Jordan three studies in Turkey) reported women seeking legal advice and shelters (one study in Saudi Arabia and Jordan four studies in Turkey) after DV. The highest proportion of women who reported to the police was in Turkey (86.6% in 2015), while the lowest was in Iraq (1.2% in 2017) and Jordan (less than 1% in 2014). Women in Lebanon, Palestine, Iran, Tunisia, and Egypt did not report to the police after DV. A mixed-method study in Jordan (Spencer et al., 2014) identified sharing information about violence with a religious leader as women’s responses to DV (see Table 3).

## Discussion

This scoping review revealed the diversity of DV against women in NA and the ME countries. The lifetime prevalence rates of DV varied widely across the region, ranging from 35% in Lebanon to 89.3% in Turkey. The range of DV in the form of physical and psychological/verbal abuse was wider in Turkey and Iran than in Jordan, Saudi Arabia, and Yemen. This finding is consistent with those of previous studies focusing in NA and the ME. A review study in 2008 about intimate partner violence in NA and the ME reported that intimate partner violence was pervasive across the region, with prevalence rates ranging from a low of 8% to a high of 65% (Boy & Kulczycki, 2008). Decker et al. (2015) found that gender-based violence against young adult women was pervasive in low and middle-income countries. However, studies that met the research criteria related to VAW in Algeria, Bahrain, Kuwait, Libya, Morocco, Sudan, Oman, Qatar, and the UAE was limited in the literature. A recent review study reported a lack of data and inconsistencies in measuring intimate partner violence in Saudi Arabia (Alhalal et al., 2019). This finding suggests that despite the improvements aimed at preventing VAW in most NA and the ME countries, DV against women seems to be an ongoing, concealed problem. This review did not find any studies that provided data on economic violence in Iran, Iraq, Palestine, or Syria, indicating a common problem of under-reporting economic and sexual violence in the region. This result is in line with the current literature. Studies reported limited data on economic VAW, especially in societies where women’s social status is low and economic violence is closely related to society’s cultural, social, and religious norms (Fawole, 2008; Haghighat, 2013; Lafta, 2008). Although economic violence results in poverty and poverty-related problems such as the increased risk of sexual exploitation, sexually transmitted diseases, and human trafficking, unfortunately, the studies on VAW have mainly focused on the prevalence of physical violence and its health consequences. This finding demonstrates a need to focus more on multiple types of DV, such as sexual and economic violence. Robust policies and educational programs within the context of structural and cultural determinants to encourage abused women to report all types of DV are suggested.

This review showed that abused women in NA and the ME countries were younger, less educated, nonworking, housewives, married to less educated husbands, witnessing, or experiencing violent behavior in the home, living in rural households, and had a low socioeconomic status. These results are in line with the literature. Well-established literature on VAW shows that young age, low educated couples, being a housewife, low socioeconomic status, alcohol or drug use, and childhood violence or witnessing violence in the family are among the factors that increase DV against women (Colorado-Yohar et al., 2016; Flood & Pease, 2009; Haj-Yahia, 2003). In contrast to Western literature, this review highlighted some culture-specific risk factors in the region. For example, the studies from Egypt and Iran reported that female circumcision, which is a pervasive cultural practice in NA and the ME countries, endogamous marriage, having a co-wife, and a male child preference increased the risk of VAW. The United Nations highlighted these pervasive traditional violent behaviors against women as priorities in achieving gender equality and empowering all women and girls (United Nations, 2020). In most patriarchal societies, younger women are dependent on their husbands and are placed at the lowest level of the hierarchy in the traditional family until they bear a male child (Haghighat, 2013; Haj-Yahia, 2003). A study by Flood and Pease (2009) indicated that attitudes towards gender roles play a significant role in perpetuating VAW in patriarchal societies. Their argument was based on women’s agreement with violence-supportive understandings of DV. It is crucial to identify suitable long-term interventions to eradicate DV among young, low educated, unemployed, and rural women.

The reported health consequences were similar to the findings of DV studies conducted in Western societies. The primary health consequences due to DV included mental health problems, pregnancy-related problems, and physical injuries. This finding is consistent with the literature. It is well described in the literature that violence negatively affects women’s physical and mental health status (Alhalal et al., 2019; Fawole, 2008;). Maxwell et al., highlighted the increased risk of having an unintended pregnancy and found that women’s experience of IPV was associated with a 30% increase in the risk of unintended pregnancy (Maxwell et al., 2017). Abused women in male-dominated societies may have a high risk of unintended pregnancy and an unmet need for family planning after violent behaviors due to their limited ability to make decisions regarding their fertility (Rahman et al., 2012). Studies have also reported a strong association between sexual intimate partner violence and depression (Al Dosary, 2016; Kamimura et al., 2016). According to the WHO (2013), abused women are almost twice as likely to experience mental health problems like depression.

It is well established in the literature that even in well-developed societies, help-seeking behaviors, particularly from formal supports such as law enforcement, are very low among women experiencing DV (Zaykowski, 2014). A similar result obtained in this review. The abused women in NA and the ME countries either did not seek help when faced violence from their husbands or partners or did not prefer to report DV to the police. Studies indicated that victims of DV are more likely to seek help from their friends and family members than legal help and severity of the injury determine the direction of the help-seeking behaviors (Akadli Ergocmen., 2013; Spencer et al., 2014; Zaykowski, 2014). Even though the studies did not include detailed information about why and how women reported to healthcare professionals, reporting to healthcare professionals was frequent in the reviewed studies. These were the main responses reported in the related literature. In the region, women’s responses toward violent behaviors were shaped by the societal attitudes. Gender norms, the responsibility to protect family honor, self-blame, embarrassment, shame, fear of being stigmatized by society as disobedient women, fear of being rejected by the family, and losing their children were reported as an excuse to accept DV (Flood & Pease, 2009; Haj-Yahia, 2003; Yount & Li, 2010). Few studies reported women seeking legal advice in Saudi Arabia, Jordan, and Turkey and location in shelters in Saudi Arabia, Jordan, and Turkey after DV (Akadli Ergocmen et al., 2013; Alan et al., 2016; Barnawi, 2017; Spencer et al., 2014). The women living in male-dominated societies do not have freedom due to their low social status, meaning that they are not employed, have a low or no education, and do not have income-generating work (McGinn & Oh, 2017). Therefore, many women are not aware of the regulations on DV against women or how to exercise these rights. Thus, activities related to increasing legal awareness is important for women living in the ME and NA countries to lower the burden of DV against women.

Moreover, women’s perspectives on violence vary according to the culture of the society in which they live, existing legal regulations, and women’s education and socioeconomic levels. Most of the women in these regions depend on their husbands for survival and are not aware of alternatives or their legal rights about DV. These obstacles prevent women from reporting husband violence to officials. Women, therefore, stay silent or prefer not to speak about the violence unless it is severe. Moreover, women in NA and the ME societies who do not follow patriarchal norms and values such as revealing family secrets, disobedience to husband or adopting the option of divorce are at higher risk of being either abused or killed (Kaur & Garg, 2008; Xavier et al., 2017). For these reasons, the findings in this review should be interpreted by taking these cultural characteristics into consideration.

### Strengths and Limitations

The current scoping review had a few limitations. First, the inclusion criterion for the language of publication could be regarded as a limitation. Due to the diversity of languages, we might have overlooked essential studies published exclusively in the local languages. Second, a limitation of this review may be the practical restrictions placed on searching electronic databases and gray literature sites. As such, the researchers may have missed some critical evidence. Nevertheless, using multiple reviewers at each stage of the review, completing inclusion criteria forms, and resolving disagreements through discussion until consensus was achieved added strength to the study. Third, the results of the review were limited to the key search terms used in the research.

## Conclusions

In this scoping review, the prevalence rates of DV against women varied widely across countries. Most studies have looked into DV in the form of physical and psychological violence; however, there is a lack of research on economic and sexual violence. The review identified the factors associated with an increased likelihood of women facing DV by a husband or intimate partner in the NA and ME region. These factors included younger women, women with lower education, witnessing or experiencing violent behavior in the home, a longer marriage duration, and a lower income level. As a result of DV, women who live in NA and the ME countries may suffer poor mental health, low health status, poor reproductive health outcomes, and homelessness. More culturally appropriate research in this field will facilitate a greater understanding of the needs and service support required for women in the NA and the ME region. Therefore, culture-sensitive research with validated tools for male-dominated societies is needed to measure DV against women. Professionals working with abused women should be aware of the cultural diversity related to DV and the variety in women’s responses to violent behaviors in NA and ME regions. In sum, it is suggested that there is a need to combat DV against women in the ME and NA countries with robust strategies focusing on men to empower gender equality in the patriarchal society.

### Critical Findings

- The prevalence rates of DV in the form of physical and psychological violence varied widely across countries in the region
- Culture-specific contributing factors such as endogamous marriage, having a co-wife, acceptability of violence by women, and a male child preference were reported as negatively associated determinants for DV
- Few studies reported women seeking legal advice and shelters
- No studies reported structural factors like perception of security forces toward abused women, the social response to VAW in countries, legal regulations to prevent VAW.
- Most of the studies were designed as a cross-sectional descriptive based on a questionnaire or survey.
- This scoping review revealed a lack of research on economic and sexual violence in the region.

### Implications for Practice, Policy, and Research

#### Practice

- Policymakers should prepare policies and educational programs to encourage abused women to report all types of DV.
- Because of the culture-specific factors, findings from this review may help professionals who care for abused women, such as health care professionals and security forces, to identify suitable interventions.

#### Policy

- There is a need to establish mass media campaigns to create awareness about legal rights in domestic/intimate partner violence.
- We suggest developing policies that support women’s empowerment within the context of structural and cultural determinants.

#### Research

- It is strongly recommended to design culture-sensitive research with validated tools for male-dominated societies to measure DV/intimate partner VAW.
- Future research should focus more on multiple DV types, such as sexual and economic violence, and determine individual and community beliefs about the acceptability of DV, gender inequalities.
- Further studies should be conducted to determine the associations of DV related health consequences and response to violence
